# Microbial global transport: An annotated selection of World Wide Web sites relevant to the topics in environmental microbiology

**DOI:** 10.1111/1758-2229.13150

**Published:** 2023-03-13

**Authors:** Lawrence P. Wackett

**Affiliations:** ^1^ McKnight Professor, Department of Biochemistry, Molecular Biology & Biophysics BioTechnology Institute, University of Minnesota St. Paul MN 55108 USA

## Global transport of microbes

### https://phys.org/news/2017‐09‐global‐microbes.html

This general article describes the issues and problems associated with human activities that move microbes around the globe at an unprecedented rate.

## Airborne transport over oceans

### https://www.nature.com/articles/s41467‐017‐00110‐9

This study looked at atmospheric transport of microbes over tropical and sub‐tropical waters. The magnitude of microbial transport was estimated to be greater than 10^21^ cells.

## Atmospheric transport of microbes

### https://agupubs.onlinelibrary.wiley.com/doi/pdf/10.1029/2011EO300001

This is a good review representing the state of knowledge on atmospheric transport of microbes as of 2011.

## Transport in the atmosphere: MicrobeWiki

### https://microbewiki.kenyon.edu/index.php/Activity_and_Transport_of_Microbes_in_the_Atmosphere

This is a relatively short but interesting overview of the presence and activities of microbes in the atmosphere.

## Microbial ecology of the atmosphere

### https://academic.oup.com/femsre/article/46/4/fuac009/6524182

Some studies have looked largely at the numbers or activities of microbes in the atmosphere. This review focused on the biogeography of the microbes, their dispersal and how anthropogenic activities affect atmospheric microbial communities.

## Dust‐associated microbial communities

### https://journals.asm.org/doi/10.1128/Spectrum.01447‐21

This comprehensive study examined microbial communities in dust samples obtained from 33 countries distributed over 6 continents.

## Airborne microbial transport over Antarctic soil

### https://researchcommons.waikato.ac.nz/bitstream/handle/10289/13245/Airblimits.pdf;jsessionid=1BD1C8DFEEC7F00E39CB39EACEA5E2B5?sequence=42

This article focused on Antarctic Dry Valleys and concluded that airborne communities of microbes are largely localized and not well‐dispersed on an inter‐continental scale.

## Microbial ecology of the planetary boundary layer

### https://www.mdpi.com/2073‐4433/11/12/1296

While some studies have looked at microbes in the stratosphere, this report focused on microbial communities found in the troposphere.

## Climate change and microbes

### https://fems‐microbiology.org/femsmicroblog‐climate‐change‐affects‐microbes/

This community science blog provides a fun look at some of the potential consequences emanating from the presence of atmospheric microbes.

## Microbial mass movements

### https://www.science.org/doi/abs/10.1126/science.aao3007

This commentary emphasizes the impacts of human activities on the spread of microbes, genes, and gene functions such as antibiotic resistance.

## Microbes and cosmic dust in the International Space Station

### https://www.hindawi.com/journals/tswj/2018/7360147/

This report described sampling for microbes on the illuminator of the International Space Station during a spacewalk. After transport to earth, DNA was amplified by PCR and sequenced. It is unclear if contamination from surface microbes might have been an issue.

## Global travelers pick up genes

### https://medicine.wustl.edu/news/global-travelers-pick-up-numerous-genes-that-promote-microbial-resistance/

This study described analyzing the gut microbiomes of international travelers. It suggested the acquisition of genes encoded by bacteria that were endemic to the region that was travelled to.

### The atmospheric microbiome

#### https://blogs.scientificamerican.com/life-unbounded/the-atmospheric-microbiome/

This short article provides a nice perspective on microbes being transported by or finding a habitat in the Earth's atmosphere.

